# Neutrophil-Lymphocyte Ratio as a Potential Biomarker for Delirium in the Intensive Care Unit

**DOI:** 10.3389/fpsyt.2021.729421

**Published:** 2021-11-29

**Authors:** Chai Lee Seo, Jin Young Park, Jaesub Park, Hesun Erin Kim, Jaehwa Cho, Jeong-Ho Seok, Jae-Jin Kim, Cheung Soo Shin, Jooyoung Oh

**Affiliations:** ^1^Department of Neuropsychiatry, Seoul National University Hospital, Seoul, South Korea; ^2^Institute of Behavioral Sciences in Medicine, Yonsei University College of Medicine, Seoul, South Korea; ^3^Department of Psychiatry, Yongin Severance Hospital, Yonsei University College of Medicine, Yongin, South Korea; ^4^Department of Psychiatry, National Health Insurance Service Ilsan Hospital, Goyang, South Korea; ^5^Department of Pulmonary and Critical Care Medicine, Gangnam Severance Hospital, Yonsei University College of Medicine, Seoul, South Korea; ^6^Department of Psychiatry, Gangnam Severance Hospital, Yonsei University College of Medicine, Seoul, South Korea; ^7^Department of Anesthesiology and Pain Medicine, Yongin Severance Hospital, Yonsei University College of Medicine, Yongin, South Korea

**Keywords:** delirium, neutrophil-lymphocyte ratio, C-reactive protein, intensive care unit, inflammatory markers

## Abstract

**Background:** Recognition and early detection of delirium in the intensive care unit (ICU) is essential to improve ICU outcomes. To date, neutrophil-lymphocyte ratio (NLR), one of inflammatory markers, has been proposed as a potential biomarker for brain disorders related to neuroinflammation. This study aimed to investigate whether NLR could be utilized in early detection of delirium in the ICU.

**Methods:** Of 10,144 patients who admitted to the ICU, 1,112 delirium patients (DE) were included in the current study. To compare among inflammatory markers, NLR, C-reactive protein (CRP), and white blood cell (WBC) counts were obtained: the mean NLR, CRP levels, and WBC counts between the initial day of ICU admission and the day of initial delirium onset within DE were examined. The inflammatory marker of 1,272 non-delirium patients (ND) were also comparatively measured as a supplement. Further comparisons included a subgroup analysis based on delirium subtypes (non-hypoactive vs. hypoactive) or admission types (elective vs. emergent).

**Results:** The NLR and CRP levels in DE increased on the day of delirium onset compared to the initial admission day. ND also showed increased CRP levels on the sixth day (the closest day to average delirium onset day among DE) of ICU admission compared to baseline, while NLR in ND did not show significant difference over time. In further analyses, the CRP level of the non-hypoactive group was more increased than that of the hypoactive group during the delirium onset. NLR, however, was more significantly increased in patients with elective admission than in those with emergent admission.

**Conclusion:** Elevation of NLR was more closely linked to the onset of delirium compared to other inflammatory markers, indicating that NLR may play a role in early detection of delirium.

## Introduction

Delirium commonly occurs among patients hospitalized in the intensive care unit (ICU). Along with the direct physiological consequences of general medical conditions, delirium is an independent predictor of poor outcomes, including prolonged ICU and hospital stay, low quality of life, and increased mortality (1–5). In this light, it is necessary to identify reliable and easily accessible biomarkers to improve the recognition and early detection of delirium (6, 7).

The underlying pathophysiology of delirium remains unclear due to the cumulative effect of multiple interconnecting results (4, 8–10). To date, several theories have been proposed to elucidate the development of delirium (11). Each proposed theory has focused on a specific mechanism of pathologic process, observational, and empirical evidence (11). The oxidative stress hypothesis asserts that a high brain oxidation metabolic rate and a low antioxidant capacity may increase damage to the brain tissue, leading to the development of persistent delirium (12). The decreased brain oxidative metabolism also results in abnormalities in various neurotransmitter systems, causing brain dysfunction and the behavioral symptoms of delirium (10). In the neurotransmitter hypothesis, the dysfunction of neurotransmitters associated with cholinergic and dopaminergic systems, such as reduced availability of acetylcholine, excess discharge of dopamine, norepinephrine, and/or glutamate, and alterations in serotonin, histamine, and/or gamma-aminobutyric acid, would lead to delirium (10, 13). In fact, some of the major triggers for cholinergic hypoactivity have long been recognized as the underlying cause of delirium (14). Imbalance and changes in neurotransmitters are also associated with neuroinflammatory processes (10, 15).

According to the neuroinflammatory hypothesis, the neuroinflammation and immune responses due to acute injury or extensive physical stress may result in delirium (11, 16). Several inflammatory markers have also been found to be related to cognitive dysfunctions. For example, neutrophil-lymphocyte ratio (NLR) and the C-reactive protein (CRP) are regarded as the underlying mediators for the connection between inflammatory markers and acute/chronic cognitive changes (17, 18). Traditionally, during the high inflammatory period, a higher CRP level is associated with a higher risk of post-operative delirium (17). According to recent studies, however, NLR can be a better predictor of mortality and outcomes in various medical conditions compared to a common infection indicator, CRP (7, 8, 19, 20). The increased NLR, a cost-effective and easily obtainable inflammatory marker, was associated with other clinical diseases that were found to be related to the inflammatory process, including cardiovascular and cerebrovascular diseases (21–24). NLR was also associated with other neuropsychiatric disorders such as Alzheimer's disease and schizophrenia, which appear to have similar clinical features of delirium, including psychotic symptoms and cognitive dysfunctions (7, 18, 25).

The previous research found that patients with delirium had higher levels of NLR than patients without delirium, suggesting that an inadequate response of the immune system and oxidative stress may play a role in the pathogenesis of delirium (7). However, the study only measured the difference between delirium and non-delirium patients. Repeated NLR measurements over time were required to provide evidence for a possible role of NLR in the course of delirium. Furthermore, the study included a relatively small number of patients. The CRP levels and WBC counts have been compared but were only measured once (26, 27), which is still remained unclear between patients with and without delirium. Therefore, it is questionable whether NLR is truly related to delirium and whether it is more useful than CRP. Since both NLR and CRP levels may serve as potential predictors and biomarkers for delirium, the present study examined the role of NLR and CRP, especially in patients with delirium in the ICU. As the primary outcome, we investigated serial changes of NLR and CRP during the course of delirium onset, while comparing the changes over time in non-delirium patients.

Furthermore, two key factors may affect the incidence and prognosis of delirium. The delirium motor subtypes exhibited different responses to treatment, length of delirium duration, and long-term outcomes (28). Emergency admission is also a risk factor for delirium in the ICU, whereas elective admission may have a lower chance of delirium onset (21). Thus, as secondary outcomes, this study also examined the changes in inflammatory markers in relation to the delirium subtypes (non-hypoactive and hypoactive motor subtypes) and admission types (emergency and elective admission).

## Materials and Methods

### Participants

**Figure 1:**
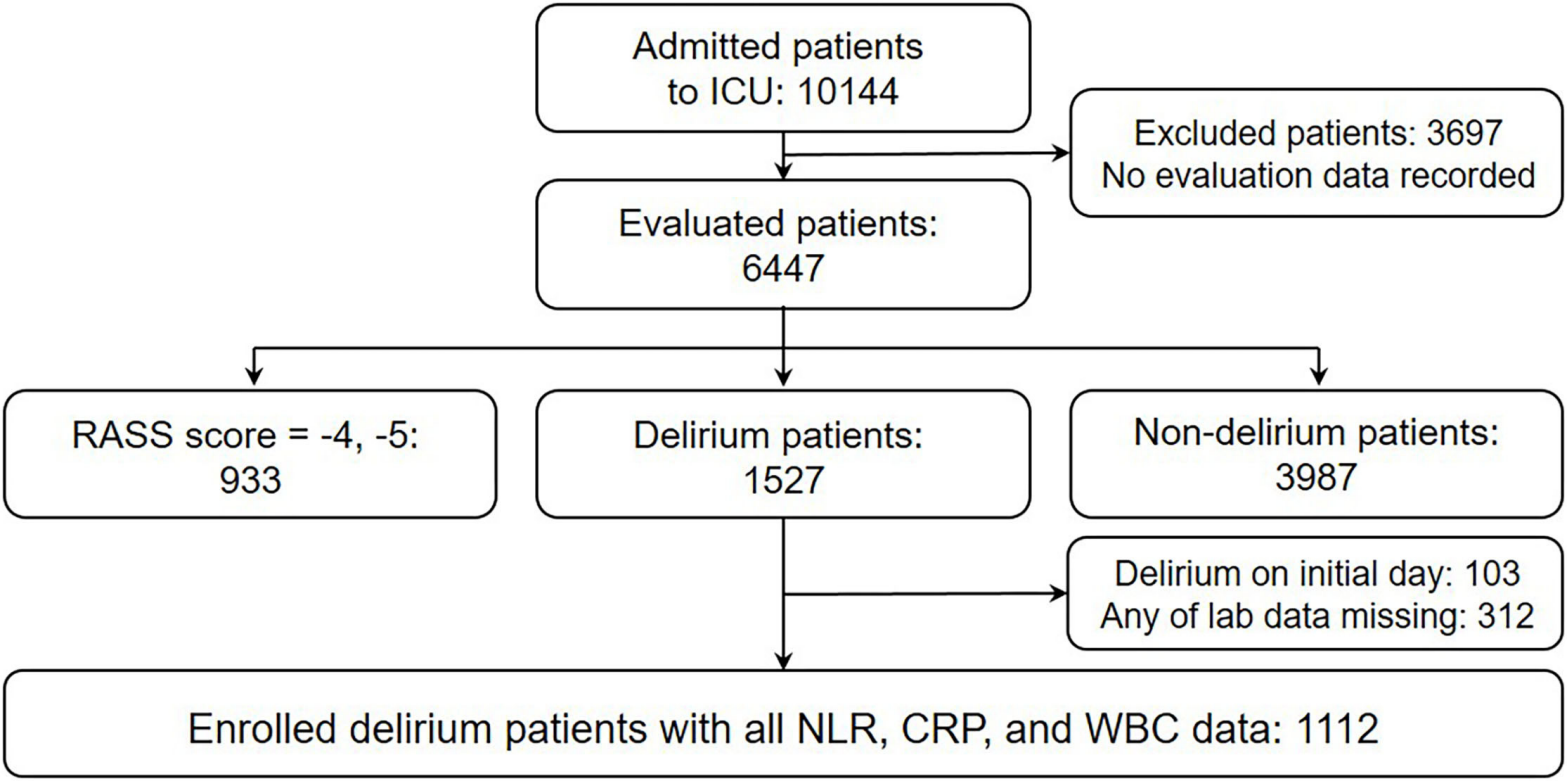
Flowchart of inclusion criteria. ICU, intensive care unit; RASS, Richmond Agitation-Sedation Scale; NLR, neutrophil-lymphocyte ratio; CRP, C-reactive protein; WBC, white blood cell.

A longitudinal observational study was conducted on patients admitted to the ICU at Gangnam Severance Hospital, a tertiary referral hospital in Seoul, Korea, between January 1, 2013 and April 30, 2020. This study was a part of the ongoing ICU Distress and Delirium Management (IDDM) project for monitoring delirium and distress among ICU patients (22). A total of 10,144 patients were admitted to the ICU during the study period, of which 1,112 patients with delirium were finally selected for this study. The exclusion criteria for the delirium group (DE) were as follows: (1) no evaluation data recorded or patient absent at the scheduled time of evaluation; (2) continuous deep sedation [Richmond Agitation-Sedation Scale (RASS) = −4 or −5]; (3) no delirium experience during the ICU stay; (4) no baseline data owing to the occurrence of delirium on the day of the initial admission; and (5) missing laboratory data of neutrophils, lymphocytes, total WBC counts, or CRP level (Figure 1). Among the 10,144 patients, the non-delirium group (ND) was initially selected all patients in neither delirious state or continuous deep sedation (RASS = −4 or −5), which were 3,987 patients. We further excluded any patient who had been discharged from ICU before the 6th ICU day—the closest day to average delirium onset day among DE. Considering the inclusion and exclusion criteria for ND above, 1,272 were included in the final analysis.

### Measures

#### Assessment of Delirium (IDDM Protocols)

All the patients in the ICU were evaluated daily by trained psychiatrists using the RASS and confusion assessment method for the ICU (CAM-ICU), except for deeply sedated patients (RASS score = −4 or −5) at around 10 am. Decisions were made on a daily basis regarding the delirium state of the patient based on the CAM-ICU results and the Diagnostic and Statistical Manual of Mental Disorders, Fifth Edition (DSM-5). All patients over the age of 19 years were evaluated daily from the time of admission to the time of discharge. However, the evaluation by the psychiatrist was not performed in patients who had a short ICU stay (<24 h) or who were not in bed during the scheduled assessment. The evaluation results for each patient were recorded using electronic medical records (EMR).

After the mental state of the patient was found to be delirious (CAM-ICU: positive), the delirium severity and subtypes were assessed using the Korean version of the Delirium Rating Scale -Revised-98 (DRS) (23, 24) and Delirium Motor Subtype Scale (DMSS) (29, 30). While the DMSS scoring requires at least two symptoms from the hyperactive or hypoactive list to meet subtype criteria, individuals meeting both criteria were considered to have a mixed subtype (29). Patients who were diagnosed with delirium, and patients without delirium during their ICU stay were selected as participants in this study. The institutional review board of Gangnam Severance Hospital, Yonsei University, approved the retrospective analyses conducted in this study. The requirement for obtaining informed consent was waived, as this study was conducted as the routine clinical process of assessing and managing delirium (IDDM).

#### Inflammatory Markers for Delirium

All laboratory results were automatically recorded in the EMR during routine clinical evaluations. The NLR, CRP (mg/L) levels and WBC (# in 10^3^/μL) counts on ICU admission day (baseline) and the day of initial delirium onset (delirium) were used in this study. The NLR was calculated by dividing the absolute neutrophil count (# in 10^3^/μL) by the absolute lymphocyte count (# in 10^3^/μL).

### Data Analysis

All data were extracted from the EMR. Demographic information such as sex, age, delirium subtypes, length of ICU stay, Acute Physiology and Chronic Health Evaluation (APACHE) II score, admission type (emergency/elective), and operation status (surgical/medical) were collected for each patient. The total WBC, neutrophil, and lymphocyte counts, and CRP levels on the day of baseline and delirium were extracted, and the NLR was calculated. The main analysis was conducted by paired *t*-tests in order to compare the mean NLR, CRP levels, and WBC counts between the day of baseline and the onset day of delirium. In addition, ND was also analyzed and presented as comparative indicators to compare the alterations over time. Paired *t*-tests were conducted on each NLR, CRP, and WBC between the day of baseline and the 6th day of the ICU—which was the closest to the mean delirium onset day (6.44 ± 8.99) in the DE.

For further analyses, patients were classified into hypoactive or non-hypoactive motor subtypes to identify inflammatory responses according to the delirium subtypes. The non-hypoactive subtype was defined to include both hyperactive and mixed types of delirium. Moreover, the patients were categorized into emergency or elective admission in order to determine the effect of admission type on delirium. By adding the time information variables, 2 (subtypes: hypoactive, non-hypoactive) × 2 (time: baseline, delirium) two-way mixed ANOVA and 2 (admission: emergency, elective) × 2 (time: baseline, delirium) two-way mixed ANOVA were conducted on the NLR, CRP, and WBC counts. Paired *t*-tests were performed as *post-hoc* tests to examine interactions in additional detail. All statistical analyses were conducted using the Statistical Package for the Social Sciences software version 26.0 for Windows (IBM Corporation, Armonk, NY, USA).

## Results

### Demographic Information

The demographic characteristics of participants were analyzed in terms of sex, age, delirium subtype, length of ICU stay, APACHE score, operation status, and admission type (Table 1). There were significant differences between DE and ND in age [*t*_(2,382)_ = 12.09, *p* < 0.001], length of ICU stay [*t*_(2,382)_ = 2.81, *p* < 0.01], and APACHE score [*t*_(2,382)_ = 58.65, *p* < 0.001]. To be specific, ND was significantly younger and less severe compared to DE. The operation status (χ^2^ = 76.01, *p* < 0.001) and admission type (χ^2^ = 184.68, *p* < 0.001) were also significantly different. DE included more patients with the operation and urgent admission. There was no significant difference in sex between the two groups.

**Table 1:** Demographic characteristics of the patients with delirium; mean (SD).

**Variables**	**DE** **(*n* = 1,112)**	**ND** **(*n*=1,272)**	**Test statistics,** ***t/* **χ^2^****
Sex, female (%)	444 (40)	487 (38)	0.672
Age	68.90 (15.13)	59.65 (21.21)	12.09^**^
DMSS, non-hypoactive (%)	552 (50)	–	–
ICU days	9.60 (10.36)	10.84 (11.08)	2.81^*^
APACHE II	21.62 (8.00)	17.31 (6.89)	58.65^**^
DRS_severity	17.78 (5.76)	–	–
DRS_diagnostic	4.89 (1.11)	–	–
Operation status, surgical (%)	678 (61)	548 (43)	76.01^**^
Admission type, emergent (%)	882 (79)	254 (20)	184.68^**^

* *p < 0.01*,

** *p < 0.001; DE, delirium group; ND, non-delirium group; DMSS, Delirium Motor Subtype Scale; ICU, intensive care unit; APACHE, Acute Physiology and Chronic Health Evaluation; DRS_severity, Delirium Rating Scale Severity Subscale; DRS_diagnostic, Delirium Rating Scale Diagnostic Subscale*.

The mean age of the delirium patients was 68.90 ± 15.13 years and 444 patients were women (40%). A total of 560 patients (50%) had hypoactive motor subtypes of delirium, whereas 388 (35%) and 164 (15%) patients had hyperactive or mixed motor subtypes, respectively. The mean length of stay in the ICU was 9.60 ± 10.36 days. The mean APACHE II score was 21.62 ± 8.00. A total of 678 patients (61%) were admitted to the ICU after surgery, whereas 434 patients (39%) were admitted for medical reasons. A total of 882 patients (79%) were admitted to the emergency department.

### Primary Outcome

#### Changes in Inflammatory Markers From the Baseline to the Onset of Delirium (DE Group)

**Figure 2:**
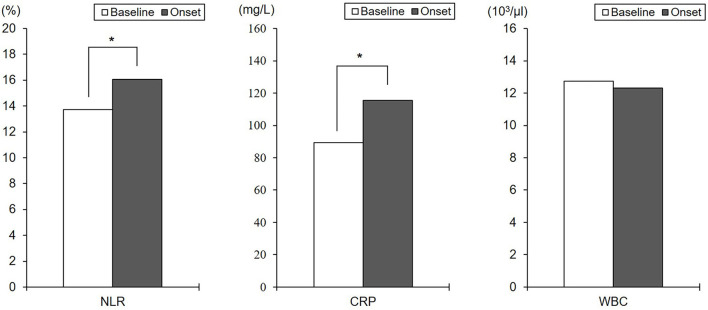
Differences between baseline and delirium onset among inflammatory markers. *p < 0.001. Baseline, the initial day of intensive care unit admission; Onset, the initial day of delirium onset; NLR, neutrophil-lymphocyte ratio; CRP, C-reactive protein; WBC, white blood cell.

There were significant differences between baseline and delirium NLR [*t*_(1,111)_ = 3.89, *p* < 0.001, *Cohen's d* = 0.13] and CRP level [*t*_(1,111)_ = 8.13, *p* < 0.001, *Cohen's d* = 0.29] in DE (Table 2). The NLR and CRP levels in DE increased on the day of delirium onset compared to the initial day of ICU admission (Figure 2). There was no significant difference in the WBC count.

**Table 2:** Differences in inflammatory markers in DE; mean (SD).

	**DE**		***t***
	**Baseline**	**Onset**	
NLR	13.72 (18.50)	16.07 (16.89)	3.89^*^
CRP	89.29 (95.55)	115.60 (84.63)	8.13^*^
WBC	12.73 (9.58)	12.32 (8.70)	1.90
Neutrophil^#^	10.30 (5.93)	10.24 (5.69)	0.32
Lymphocyte^#^	1.24 (1.07)	0.91 (0.60)	10.86^*^

* *p < 0.001; DE, delirium group; NLR, neutrophil-lymphocyte ratio; CRP, C-reactive protein; WBC, white blood cell; Neutrophil^#^. Neutrophil counts; Lymphocyte^#^. Lymphocyte counts*.

#### Changes in Inflammatory Markers From the Baseline to the 6th ICU Day (ND Group)

In terms of comparative indicators, there was no significant difference in NLR level in ND whereas CRP [*t*_(755)_ = 9.17, *p* < 0.01] and WBC [*t*_(1,256)_ = 63.76, *p* < 0.001] showed significant differences between the 1st and the 6th day of ICU (Table 3). CRP was increased over time meanwhile WBC was decreased in ND. There was no significant change in NLR in the absence of delirium.

**Table 3:** Differences in inflammatory markers in ND; mean (SD).

	**ND**		***t***
	**1st day**	**6th day**	
NLR	14.64 (24.63)	14.76 (29.63)	0.11
CRP	96.46 (98.53)	109.67 (83.52)	2.65^*^
WBC	13.16 (8.22)	10.81 (5.68)	10.45^**^
Neutrophil^#^	10.76 (6.67)	8.69 (4.76)	10.79^**^
Lymphocyte^#^	1.49 (1.61)	1.00 (0.74)	10.25^**^

* *p < 0.01*,

** *p < 0.001; ND, non-delirium group; NLR, neutrophil-lymphocyte ratio; CRP, C-reactive protein; WBC, white blood cell; Neutrophil^#^, Neutrophil counts; Lymphocyte^#^, Lymphocyte counts*.

### Secondary Outcome

#### Changes in Inflammatory Markers Considering Delirium Motor Subtypes (Non-hypoactive Subtype vs. Hypoactive Subtype)

Further analyses were conducted by dividing into subgroups (non-hypoactive motor subtype vs. hypoactive motor subtype) in order to better understand the changes in NLR and CRP in DE. The demographic information and clinical characteristics of each delirium motor subgroup were analyzed (Supplementary Table 1). The two groups generally showed similar demographics (e.g., age and sex) and clinical characteristics (e.g., APACHE II score, operation status, and admission type), except for delirium rating scales and the length of ICU stay. There were significant group differences in delirium ratings, including both severity [*t*_(1,110)_ = 4.19, *p* < 0.01] and diagnostic subscales [*t*_(1,110)_ = 2.66, *p* < 0.05]. The non-hypoactive group scored higher on delirium scales, which means that the non-hypoactive group reported severe symptoms of delirium (e.g., perception, affect, attention, and psychomotor ability). However, the length of ICU stay was significantly longer in the hypoactive group.

**Figure 3:**
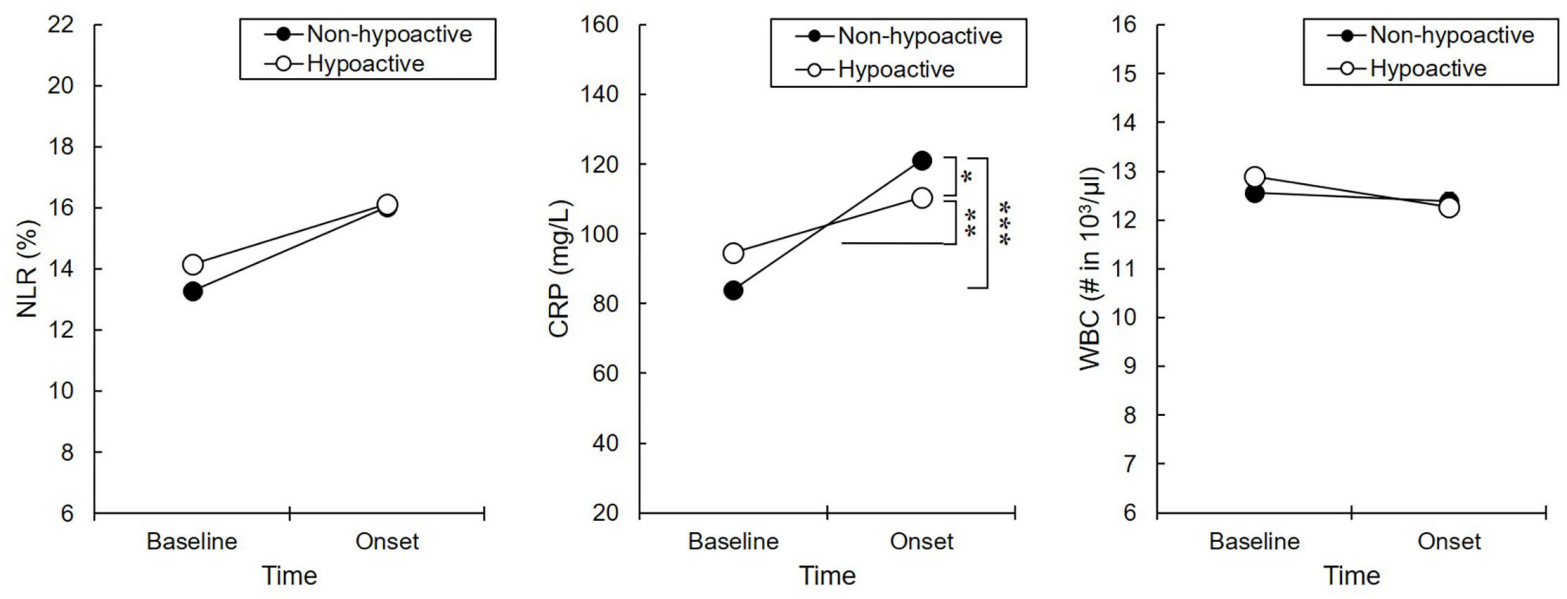
Inflammatory responses between delirium subtypes. *p < 0.05, **p < 0.01, ***p < 0.001. Non-hypoactive, non-hypoactive motor subtype; Hypoactive, hypoactive motor subtype; Baseline, the initial day of intensive care unit admission; Onset, the initial day of delirium onset; NLR, neutrophil-lymphocyte ratio; CRP, C-reactive protein; WBC, white blood cell. The CRP level in the non-hypoactive group was significantly increased than that in the hypoactive group during delirium onset.

Only CRP had a significant two-way (group by time) interaction effect [*F*_(1,1110)_ = 11.23, *p* < 0.01]. In detail, the CRP level in the non-hypoactive group was significantly increased than that in the hypoactive group during delirium onset (Figure 3). The main effect of time was also significant as the CRP level increased in delirium onset compared to baseline in both subtypes. In the *post-hoc* analysis, there was no significant group difference in the NLR or WBC count.

#### Changes in Inflammatory Markers According to Admission Types (Emergency Admission vs. Elective Admission)

Demographic information and clinical characteristics between admission types were analyzed (Supplementary Table 2). The overall demographic information and clinical characteristics were statistically not significant; however, only the APACHE II score showed a significant difference [*t*_(1,110)_ = 3.23, *p* < 0.001]. Individuals with emergency admission had higher APACHE II scores than those with elective admission.

**Figure 4:**
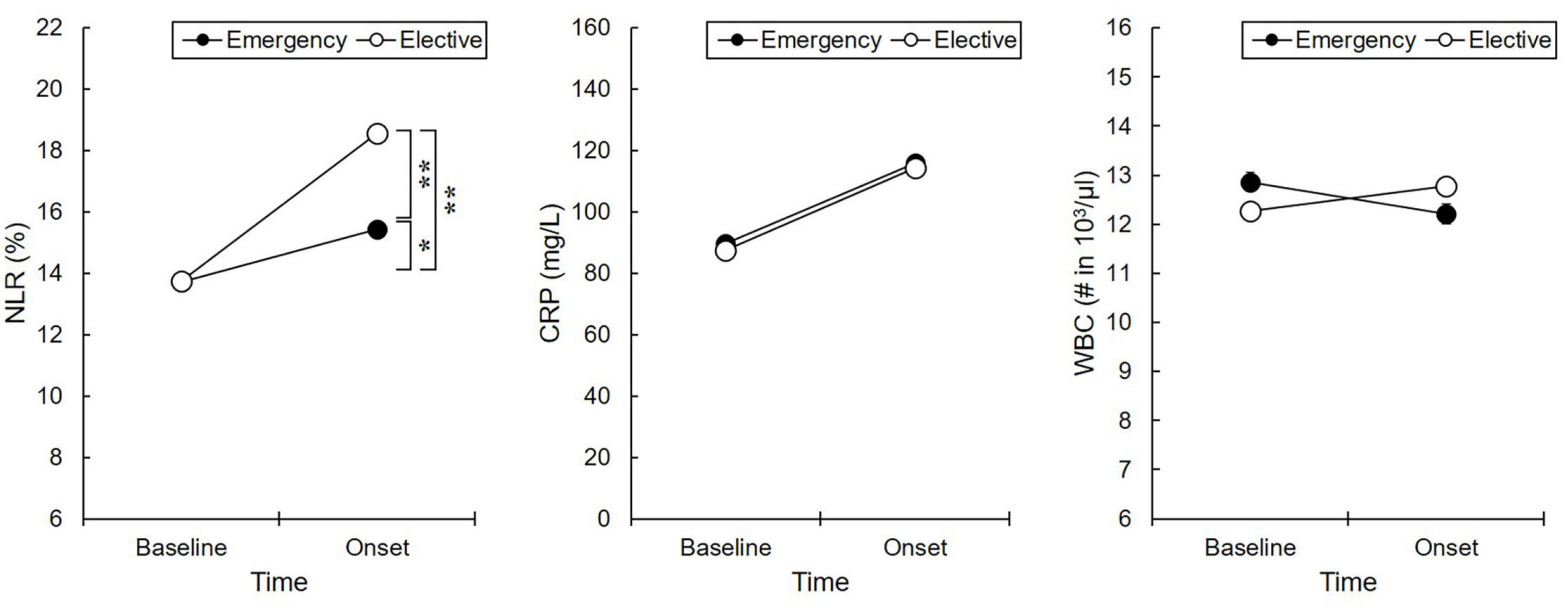
Inflammatory responses between admission status. *p < 0.05 and **p < 0.01. Baseline, the initial day of intensive care unit admission; Onset, the initial day of delirium onset; Emergency, emergency admission; Elective, elective admission; NLR, neutrophil-lymphocyte ratio; CRP, C-reactive protein; WBC, white blood cell. The NLR level in patients with elective admission was significantly increased than those with emergency admission.

The two-way (group by time) interaction effect on the NLR [*F*_(1,1110)_ = 4.28, *p* < 0.05] and WBC count [*F*_(1,1110)_ = 4.68, *p* < 0.05] were significant. In the *post-hoc* analysis, patients with elective admission showed significantly higher NLR levels than those with emergency admission, whereas the NLR between the groups was not significantly different at the baseline (Figure 4). In the *post-hoc* analysis, there was no significant group difference with respect to WBC counts.

## Discussion

**Figure 5:**
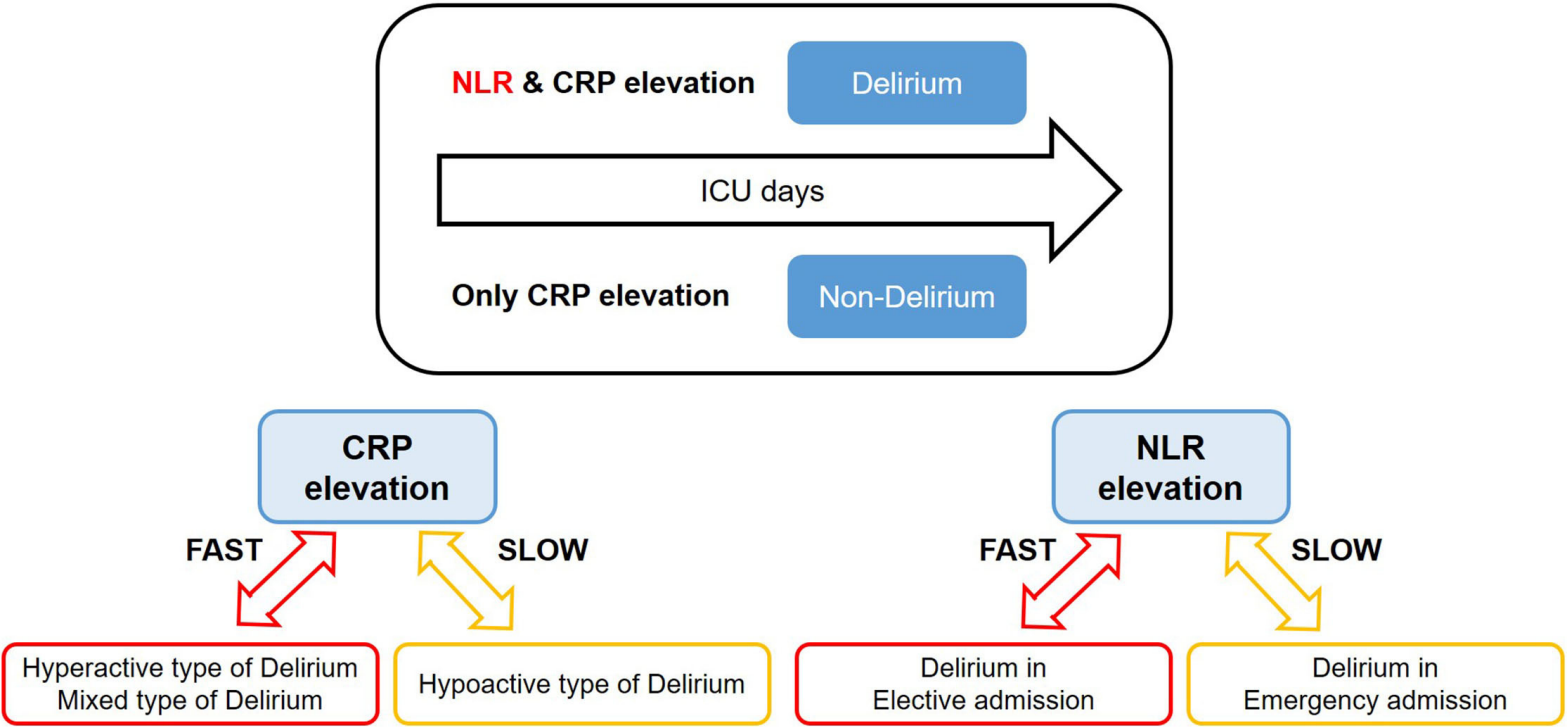
Summary of the study results. NLR, neutrophil-lymphocyte ratio; CRP, C-reactive protein. This figure shows the overall results of our study. Delirium group showed increases in both NLR and CRP over time, while non-delirium group only showed the CRP elevation. Especially, CRP was abruptly increased in delirium patients with non-hypoactive motor subtype compared to the patients with hypoactive motor subtype. NLR was increased fast in delirium patients with elective admission compared to the delirium patients with emergent admission.

This study found that the NLR and CRP levels increased on the day of delirium onset compared to those on the initial day of ICU admission. This finding indicates that alterations in inflammatory responses, both NLR and CRP, may play a role in the occurrence of delirium. Increased NLR levels were the unique response only in the delirium group whereas CRP levels were elevated over time both in delirium and non-delirium groups. This can be interpreted as the NLR may be the more effective inflammatory marker in early detection of delirium occurrence, compared to CRP (Figure 5). This result is in line with recent studies using NLR for prediction of delirium (31, 32).

In order to determine which inflammatory responses could be utilized under certain circumstances, this study conducted further analyses by splitting delirium patients into (1) delirium subtypes and (2) admission types. The non-hypoactive delirium motor subgroup had an elevated CRP level compared to the hypoactive subgroup during delirium onset, although no significant difference between the two subgroups at baseline was observed. The NLR differed statistically depending on the subgroups of emergency and elective admission. This study revealed that the NLR was high in individuals with elective admission during delirium occurrence; the two subgroups did not show any difference in the NLR on the initial day of ICU admission.

This study suggests that NLR can be potential biomarkers for delirium. While activation of the immune system is a prominent feature of the physical conditions associated with delirium, NLR, the integrated marker of two inflammatory components, would be useful for early detection (7). Neutrophils are one of the first-line defenses during inflammation; once activated, they release reactive oxygen species, myeloperoxidase, and proteolytic enzymes in an attempt to destroy pathogens or damaged cells (33, 34). Lymphocytes are also responsible for regulating an appropriate inflammatory response. Although a decrease in the lymphocyte count is a normal response during periods of acute stress, a chronic decrease in the lymphocyte count might lead to a detrimental inflammatory state and ultimately result in poor clinical outcomes (35, 36). In the balance between neutrophils and lymphocytes, NLR is a readily available biomarker that conveys important information regarding the complex inflammatory activity and can be easily obtained (7).

According to the results of this study, CRP might be used as an early-recognition biomarker for hyperactive and mixed motor delirium subtypes. CRP is related to increased physical activity, even in the attention deficit and hyperactivity disorder pathogenesis, mediated through changes in cytokines and neurotransmitters. When CRP levels increase rapidly during the monitoring of ICU patients, it would be helpful to start and optimize non-pharmacological interventions (e.g., trying to make their surroundings more familiar, and providing appropriate stimulation to maintain orientation, memory and thinking skills) before they show behavioral problems during ICU stay. Preventive antipsychotics are not recommended currently, however, some meta-analyses showed the limited possibility that antipsychotics may have a preventive role in patients with post-operative delirium (37, 38).

Patients with delirium after elective admission had a faster increase pattern in NLR than delirium patients with emergent admission. In other words, although we did not compare with the changes in non-delirium patients, it seems difficult to use NLR as a marker for detect delirious changes in patients with emergent admission. Patients with emergency admission have a higher risk of complications and may be influenced by traumatic or neoplastic changes, which may necessitate urgent interventions (39). Considering the NLR was not effective in showing the delirious changes in those patients; therefore, it seems necessary to explore other indicators different from NLR for patients with emergency admission. However, as shown in our data, patients with elective admission had lower APACHE II scores than the emergency patients, which may overlook the clinical risks of delirium. For these patients, tracking abrupt changes in the NLR were found to be more related to the delirium.

This study has some limitations in terms of missing causative associations and potential confounders. Since this is an observational study that monitored real clinical situations retrospectively, the causality between inflammatory markers and the occurrence of delirium is unclear. Since delirium can be affected by numerous factors such as existing medical conditions, clinical diagnosis, use of ventilators, former neurocognitive impairment and so on, further research is necessary to replicate the results with a fully controlled and designed experiment. Secondly, the distribution of the lab data was moderately skewed (skewness = 0.73). Despite of non-normality of the data, we still assumed that the mean of a large sample size in the current study would be sufficient for predicting the characteristics of a population accurately, according to the central limit theorem. Future study, however, could eliminate the outliers so that the data is close enough to the normal distribution. Thirdly, the onset day of delirium was also non-normally distributed; the median (3rd day of ICU admission) was lesser than the mean (6th day of ICU admission). However, we used the mean day for comparison of ND group. Because, if median was used, all patients who had stayed for 3 days or more would have been included in the ND group, then the length of ICU stays in ND group became significantly shorter than DE group, making it difficult to compare under similar conditions. Since the current study retrospectively examined the overall trend of inflammatory responses, there is a limitation in that non-delirium patients were not ideally matched as a control group. Due to these limitations, the usefulness of NLR for delirium detection that occurs very early in ICU admission has not been sufficiently verified. This study was conducted in only one tertiary hospital using heterogeneous patients; therefore, it is difficult to generalize the results. Lastly, due to strict inclusion and exclusion criteria for both delirium and non-delirium patients such as the presence of lab data, the number of selected participants was somewhat lower than the incidence of delirium in the ICU. There also might be a few missed diagnoses of delirium at night since the daily assessment was only conducted in the morning. However, our findings may have some implications for patients of other institutions with similar severe conditions, as we examined serial changes within the same patient. A multi-center study using a wide variety of potential variables may overcome these limitations.

## Conclusions

Despite these limitations, to the best of our knowledge, this is the first study to evaluate NLR and CRP in pre- and post-delirium onset in a large number of patients. This study shows that changes in NLR can be more efficient in early detecting overall delirium, compared to CRP. Our study also revealed that changes in inflammatory markers might be useful in predicting delirium, while considering subtype and urgency of admission. The NLR and CRP can be useful in elaborating existing delirium prediction models, such as the prediction of delirium in the ICU model (PRE-DELIRIC) (40), and can also be utilized as key features in machine learning-based future prediction models. These biomarkers may aid in risk stratification, diagnosis, and monitoring of delirium, and may help to find an effective treatment.

## Data Availability Statement

The original contributions presented in the study are included in the article/Supplementary Material, further inquiries can be directed to the corresponding author/s.

## Ethics Statement

The studies involving human participants were reviewed and approved by the Institutional Review Board of Gangnam Severance Hospital, Yonsei University. Written informed consent for participation was not required for this study in accordance with the national legislation and the institutional requirements.

## Author Contributions

CS and JO designed and conceptualized the study, conducted the literature search, interpreted the data, and drafted and revised the manuscript. JYP, JP, JC, J-HS, J-JK, CS, and JO collected the data. CS and HK analyzed the data. All authors contributed to the article and approved the submitted version.

## Funding

This research was supported by the National Research Foundation of Korea (NRF) grant funded by the Korea government (MSIT) (No. 2020R1C1C1007440).

## Conflict of Interest

The authors declare that the research was conducted in the absence of any commercial or financial relationships that could be construed as a potential conflict of interest.

## Publisher's Note

All claims expressed in this article are solely those of the authors and do not necessarily represent those of their affiliated organizations, or those of the publisher, the editors and the reviewers. Any product that may be evaluated in this article, or claim that may be made by its manufacturer, is not guaranteed or endorsed by the publisher.
