# Supplementary material for: Neutrophil-Lymphocyte Ratio as a Potential Biomarker for Delirium in the Intensive Care Unit
Source: Front Psychiatry. 2021 Nov 29;12:729421. doi: 10.3389/fpsyt.2021.729421 (PMC8667224; doi:10.3389/fpsyt.2021.729421)
Supplement: Supplementary file 1 [file Table_1.DOCX]

**Table S1** Demographic characteristics for non-hypoactive and hypoactive delirium groups; mean (SD).

| **Variables** | **Group** | | | | **Test Statistics**  ***(t/χ2)*** | | ***p*-value** |
| --- | --- | --- | --- | --- | --- | --- | --- |
|  | **Non-hypoactive**  **(n = 552)** | | **Hypoactive**  **(n = 560)** | |  |  |  |
| Sex, female (%) | 211 | (38) | 233 | (42) | 1.33 |  | 0.250 |
| Age | 68.16 | (14.92) | 69.63 | (15.31) | 1.62 |  | 0.105 |
| ICU days | 8.64 | (9.99) | 10.53 | (10.63) | 3.05 | * | 0.002 |
| APACHE II | 21.18 | (8.10) | 22.11 | (7.87) | 1.69 |  | 0.092 |
| DRS_severity | 18.50 | (5.71) | 17.07 | (5.72) | 4.19 | ** | < 0.001 |
| DRS_diagnostic | 4.98 | (1.19) | 4.80 | (1.01) | 2.66 | * | 0.008 |
| Operation status, postoperative (%) | 325 | (59) | 353 | (63) | 2.02 |  | 0.155 |
| Admission type, emergency (%) | 435 | (79) | 447 | (80) | 0.18 |  | 0.675 |

**Notes:** ** *p* < 0.001, * *p* < 0.01.

**Abbreviations:** Non-hypoactive, non-hypoactive delirium motor subtype; Hypoactive, hypoactive delirium motor subtype; ICU, intensive care unit; APACHE, Acute Physiology and Chronic Health Evaluation; DRS_severity, Delirium Rating Scale Severity Subscale; DRS_diagnostic, Delirium Rating Scale Diagnostic Subscale; *t*, independent *t*-test; *χ2*, chi-square test.

**Table S2** Demographic characteristics for emergency and elective admission types; mean (SD).

|  | **Group** | | | | **Test Statistics**  ***(t/χ2)*** | | ***p-*value** |
| --- | --- | --- | --- | --- | --- | --- | --- |
| **Variables** | **Emergency**  **(n = 882)** | | **Elective**  **(n = 230)** | |  |  |  |
| Sex, female (%) | 357 | (40) | 87 | (38) | 0.53 |  | 0.465 |
| Age | 68.80 | (15.71) | 69.26 | (12.67) | 0.41 |  | 0.686 |
| DMSS, non-hypoactive (%) | 435 | (49) | 117 | (51) | 0.18 |  | 0.675 |
| ICU days | 9.88 | (10.46) | 8.50 | (9.91) | 1.80 |  | 0.073 |
| APACHE II | 22.09 | (8.20) | 19.85 | (6.90) | 3.23 | * | < 0.001 |
| DRS_severity | 17.81 | (5.72) | 17.66 | (5.91) | 0.35 |  | 0.728 |
| DRS_diagnostic | 4.87 | (1.10) | 4.94 | (1.15) | 0.86 |  | 0.391 |
| Operation status, postoperative (%) | 542 | (61) | 136 | (59) | 0.41 |  | 0.520 |

**Notes:** * *p* < 0.001.

**Abbreviations:** Emergency, emergency admission; Elective, elective admission; DMSS, Delirium Motor Subtype Scale; Baseline, the initial day of intensive care unit admission; Delirium, the initial day of delirium onset; NLR, neutrophil-lymphocyte ratio; CRP, C-reactive protein; WBC, white blood cell; *t,* independent *t*-test; *χ2,* chi-square test.
